# An Artificial Intelligence Algorithm With 24-h Holter Monitoring for the Identification of Occult Atrial Fibrillation During Sinus Rhythm

**DOI:** 10.3389/fcvm.2022.906780

**Published:** 2022-07-06

**Authors:** Ju Youn Kim, Kyung Geun Kim, Yunwon Tae, Mineok Chang, Seung-Jung Park, Kyoung-Min Park, Young Keun On, June Soo Kim, Yeha Lee, Sung-Won Jang

**Affiliations:** ^1^Division of Cardiology, Department of Internal Medicine, Samsung Medical Center, Sungkyunkwan University School of Medicine, Heart Vascular Stroke Institute, Seoul, South Korea; ^2^VUNO Inc., Seoul, South Korea; ^3^Division of Cardiology, Department of Internal Medicine, College of Medicine, Eunpyeong St. Mary's Hospital, The Catholic University of Korea, Seoul, South Korea

**Keywords:** atrial fibrillation, 24-h Holter monitoring, artificial intelligence, continuous ambulatory rhythm monitoring, supraventricular ectopy

## Abstract

**Background:**

Subclinical atrial fibrillation (AF) is one of the pathogeneses of embolic stroke. Detection of occult AF and providing proper anticoagulant treatment is an important way to prevent stroke recurrence. The purpose of this study was to determine whether an artificial intelligence (AI) model can assess occult AF using 24-h Holter monitoring during normal sinus rhythm.

**Methods:**

This study is a retrospective cohort study that included those who underwent Holter monitoring. The primary outcome was identifying patients with AF analyzed with an AI model using 24-h Holter monitoring without AF documentation. We trained the AI using a Holter monitor, including supraventricular ectopy (SVE) events (setting 1) and excluding SVE events (setting 2). Additionally, we performed comparisons using the SVE burden recorded in Holter annotation data.

**Results:**

The area under the receiver operating characteristics curve (AUROC) of setting 1 was 0.85 (0.83–0.87) and that of setting 2 was 0.84 (0.82–0.86). The AUROC of the SVE burden with Holter annotation data was 0.73. According to the diurnal period, the AUROCs for daytime were 0.83 (0.78–0.88) for setting 1 and 0.83 (0.78–0.88) for setting 2, respectively, while those for nighttime were 0.85 (0.82–0.88) for setting 1 and 0.85 (0.80–0.90) for setting 2.

**Conclusion:**

We have demonstrated that an AI can identify occult paroxysmal AF using 24-h continuous ambulatory Holter monitoring during sinus rhythm. The performance of our AI model outperformed the use of SVE burden in the Holter exam to identify paroxysmal AF. According to the diurnal period, nighttime recordings showed more favorable performance compared to daytime recordings.

## Introduction

Atrial fibrillation (AF) is common and has shown a progressive increase in prevalence over time (1, 2). AF is associated with increased risks of stroke and systemic embolism (2). The diagnostic criteria for AF is standard 12-lead electrocardiography (ECG) documentation or a single-lead ECG tracing of >30 s according to the current guideline (3). However, screening of AF remains challenging, and underdiagnosis is common in patients with paroxysmal AF (pAF) due to their high prevalence of asymptomatic AF. Subclinical AF is one of the pathogeneses of embolic stroke of an undetermined source (ESUS) (4). AF accounts for about 8–15% of ESUS cases, (4, 5) and these patients need anticoagulation therapy instead of aspirin treatment. However, empirical use of anticoagulation without documentation of AF did not show superiority to aspirin in preventing recurrent stroke even when using direct oral anticoagulants (6, 7). Therefore, aspirin remains the standard antithrombotic treatment in the absence of documented AF (8); nevertheless, the annual recurrent stroke rate on standard antithrombotic treatment is ~5% in ESUS patients (9).

In this sense, the detection of occult AF and provision of proper anticoagulant treatment are key strategies to prevent stroke recurrence. Prolonged ambulatory rhythm monitoring, including by non-invasive event-triggered recording, hospital telemetry monitoring, continuous ECG patch monitoring, or implantable cardiac monitoring, is recommended following ESUS (10). Recently, several mobile health technologies have been developed using smartphones or watches. In asymptomatic patients, continuous monitoring increases the diagnostic yield rather than opportunistic screening. However, implantable cardiac monitors are invasive, and their cost-effectiveness should also be considered. To find out whether the substrate for AF relates to anatomical, structural, or functional changes is one of the supportive findings in highly suspected AF cases (11).

To increase the diagnostic rate of ECG monitoring and its cost-effectiveness, it is necessary to choose a patient who is expected to have AF. This study used a deep learning model to predict occult AF in patients who showed normal sinus rhythm during 24-h Holter monitoring.

## Methods

### Study Population

This study was a retrospective cohort study that included consecutive patients aged ≥18 years who underwent 24-h ambulatory Holter monitoring from August 2019 to June 2020. We excluded patients with AF or a paced rhythm recorded during the Holter exam. All exams involved acquiring three channels (channel 1, modified V5; channel 2, modified V1; channel 3, lead III) with the SEER™ 1000 Holter recorder (GE Healthcare, Chicago, IL, USA). A raw wave signal was exported in MIT format using the CardioDay Holter ECG software (GE Healthcare, Chicago, IL, USA). This study was approved by the local Institutional Review Board (IRB), South Korea (IRB no. XC20REDE0135).

### Data Collection

The study cohort was classified into two groups. The first was an AF group with at least one atrial tachyarrhythmia clinically documented by 12-lead ECG or previous 24-h Holter monitoring identified in our electronic health records system from 2009 to 2020. Atrial tachyarrhythmias include AF, atrial flutter, and atrial tachycardia. The second group was a control group with no history of clinically documented atrial tachyarrhythmia. We defined the index time as the day when pAF was diagnosed with any ECG modality. We included the Holter exam findings from 1 year before the index time in the AF group.

**Figure 1:**
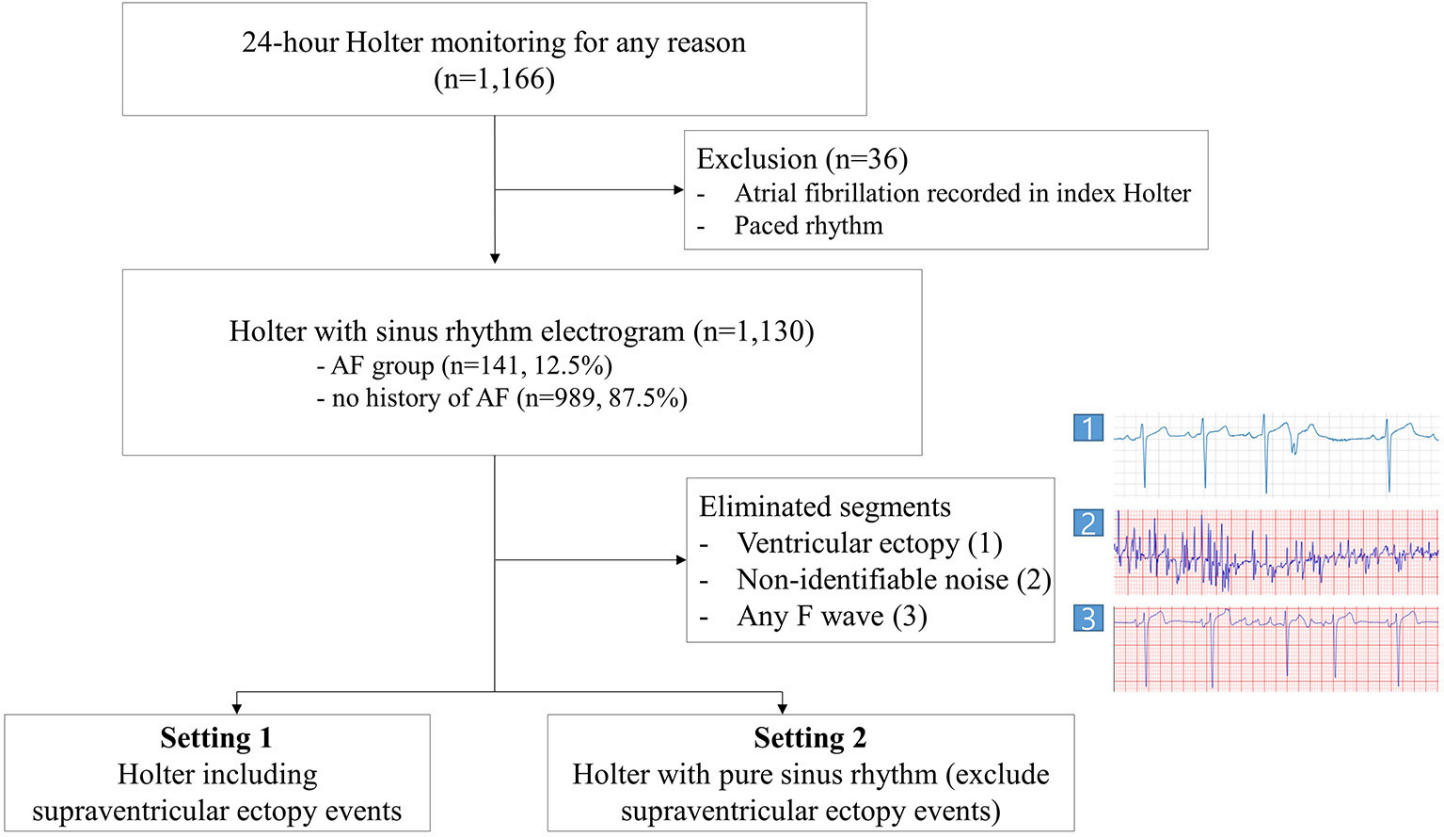
Flow chart.

Before the analysis, the electrograms annotated as an event by the Holter ECG software were extracted. These events were classified as supraventricular ectopy (SVE), ventricular ectopy, or other. Each ventricular ectopy section filtered by the Holter ECG software was eliminated from the analysis to remove the influence of T-wave turbulence following ventricular ectopy. Noise-filtering was performed for all electrograms, specifically any ECG with non-identifiable noise recorded was eliminated using a CNN based AI algorithm and the physician's discretion. We implemented reannotation for the rest of the SVE events by an electrophysiologist to eliminate any F wave to prevent overfitting. To make annotation process most efficient for electrophysiologists, we have divided the 24-h long electrogram recordings into 7-s segments making all eliminated events containing ventricular or SVE segments 7-s long. 3.5 s forward and backward based on the ectopic beat were extracted to remove all the annotated events including couplet ectopic beats. Next, we carried out three different settings for data analysis. As the first setting, we analyzed the rest of all data, including SVE events. Second, we excluded SVE events read by the Holter software and performed training using only sinus rhythm electrogram. Finally, we compared the performance of our AI model against the SVE burden-based estimator derived from Holter software annotation data. The SVE burden-based estimator computes the ratio of SVE event segments in the Holter monitor data as the pAF probability (Figure 1).

**Figure 2:**
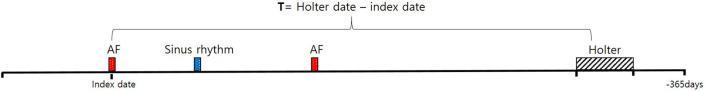
Timeframe of index date and Holter exam.

As an additional analysis, we divided the ECG segments according to the recording time to assess the difference in detection rate between daytime and nighttime with diurnal periods. Here, nighttime was defined as 22:00–07:00 with fixed times (12). Furthermore, we compared AI detection rates according to the differences between the index date and a Holter exam date cutoff (T) of 3, 6, 12, or 36 months (T = the Holter exam date–the index date of when AF was diagnosed) to assess the relationship between the information intensity of the deep learning model and the elapsed time from the initial diagnosis (Figure 2). With T as a threshold, two subgroups, where one included patients with the elapsed time being greater than T and another included patients with the elapsed time being less than T, were defined. As we moved the cutoff through from 3 to 6, 12, and 36 months, we measured the sensitivity of the AI model.

In developing the AI model, we validated the model performance with 5-fold cross-validation. All patients were randomly assigned to one of the five folds. This way, 4 of the 5 folds were used during training, and the remaining 1 was used to evaluate the model's performance. Evaluation process is done 5 times so that model performance is evaluated on all 5 groups (13). Note that, to prevent overfitting, we performed a random split at the patient level so that the data associated with a single patient in the testing set were not seen before.

### Outcomes

The primary study outcome was to identify patients with AF with the presence of occult atrial tachyarrhythmia (pAF) analyzed with our AI model using 24-h Holter monitoring without AF documentation. We created a receiver operating characteristic (ROC) curve and assessed the area under the ROC curve (AUROC), sensitivity, specificity, F1 score, and F2 score (14). We established two deep learning model; the first model included SVE events and the other included only normal sinus rhythm Holter electrograms, excluding those with ectopy events.

### Overview of the AI Model

The length of the data recorded using Holter monitors is usually too long to be considered by deep learning models because most existing neural network architectures are unsuited for processing such lengthy data. We therefore divided the 24-h-long ECG data into many 7-s segments to overcome this difficulty. Additionally, we divided the training process into two steps so that the model used in the first step learned to detect pAF segment-wise, where the second step learned to detect pAF patient-wise. In the first step of the two-step learning process, we used a convolutional neural network (CNN) architecture followed by fully connected layers. The CNN architecture consisted of three residual blocks, where each block had two sets of modules of convolutional layer followed by batch normalization and rectified linear unit layers. Since the Holter data were recorded using three leads (III, V1, and V5), ECG segments were formatted into a 3 × 896-size matrix. The size of each row represents the time axis of a 7-s signal recorded in a 128-Hz sampling rate. In order to gain robustness with respect to random noise in the ECG signals, we first Z-normalized each ECG segments and applied short-time Fourier transformation (STFT) (15) with a window size of 50 prior to the convolutional filters. At this phase of training, we labeled the ECG segment-wise such that ECG segments were labeled true if they were from accurate pAF recordings.

**Figure 3:**
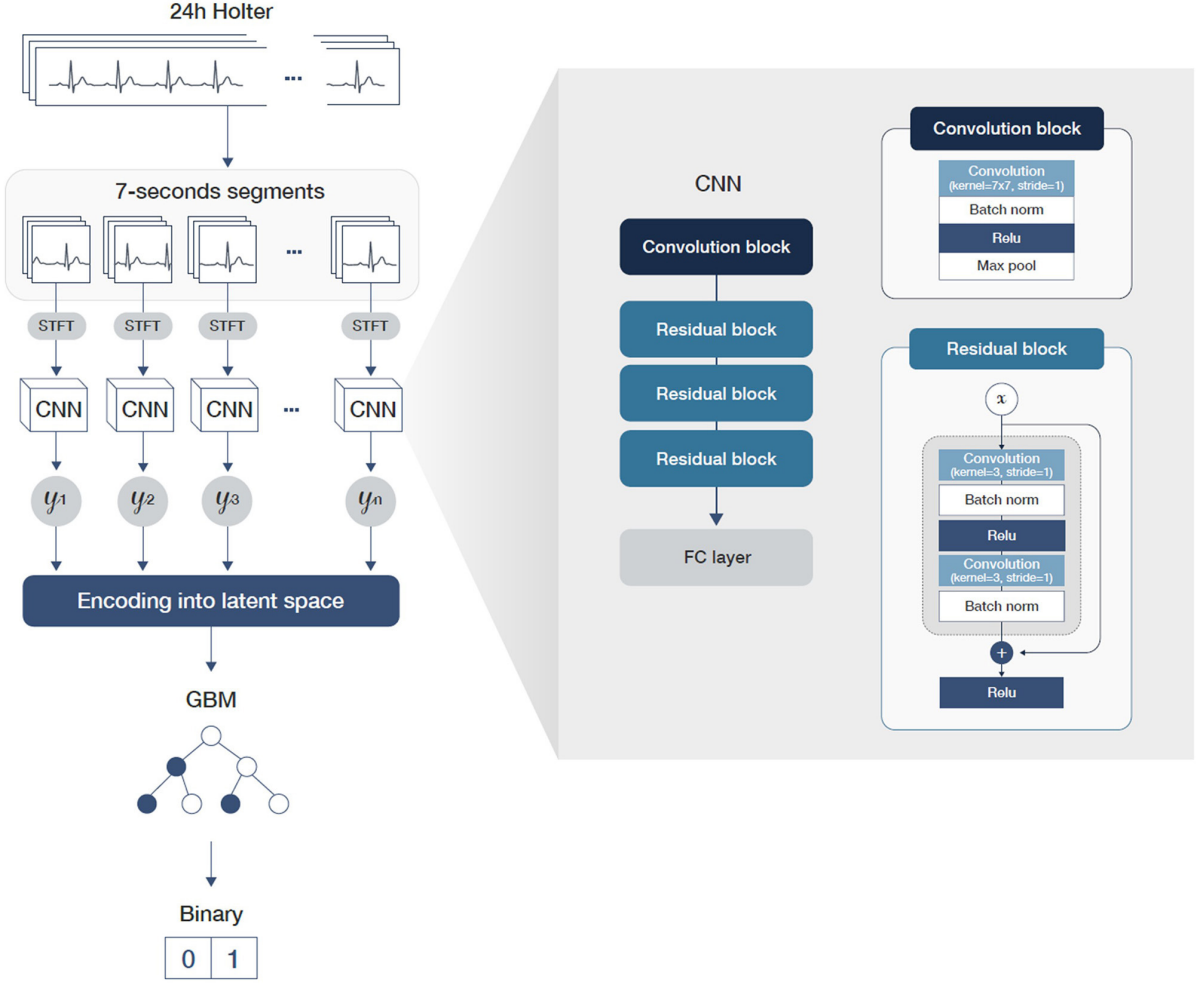
A graphical workflow and architecture of the full model including detailed parameters of the convolution and residual blocks.

In the second stage of the training step, each ECG segment was encoded using the CNN model trained in the first step and concatenated in a temporal order. As a result, the Holter recordings were mapped into the latent space of lower dimensions, making it possible for a model to consider every available information of the 24-h recordings, including time-evolving information. In this step, we trained a gradient-boosting machine (GBM) with these dimension-reduced datasets. Specifically, we used LightGBM (16) for the implementation and BOHB (17) for hyper-parameter tuning. For implementation of neural network, we used Python language with PyTorch (version 1.11.0) as deep learning library. For hyperparameters, we used learning rate of 0.0001 with batch size of 1,024. The training process was done on 8 NVIDIA GeForce RTX 3090 GPUs. Figure 3 shows the workflow and architecture of the full model.

### Statistical Analysis

Statistical analysis was performed using the Statistical Package for the Social Sciences version 27.0 (IBM Corporation, Armonk, NY, USA). Continuous variables were compared using an unpaired *t*-test or the Wilcoxon rank-sum test, while categorical variables were compared using the chi-squared test or Fisher's exact test, as appropriate. We assessed the AUC using the ROC curve. For the performance metrics of the AI model, we computed 95% confidence interval of the experiments done on 5-fold cross validation datasets. Additionally, we computed 95% confidence interval of performance metrics of SVE-burden based estimator bootstrapping 1,000 times.

## Results

### Baseline Characteristics

In total, 1,166 consecutive patients were included in this study. Among these, 36 patients who showed AF or a paced rhythm during the Holter exam were excluded. Meanwhile, 141 had pAF according to the medical records. The mean age was 61.2 ± 17.1 years at the date of the Holter exam, and 558 (49.4%) participants were male. The mean duration of Holter monitoring included in the analysis was 877.74 min, and a total of 8,501,538 segments were included for analysis. The average time from AF diagnosis to the time of Holter monitoring was 2,040 days (range, −311–43,971 days).

### AI Model

**Figure 4:**
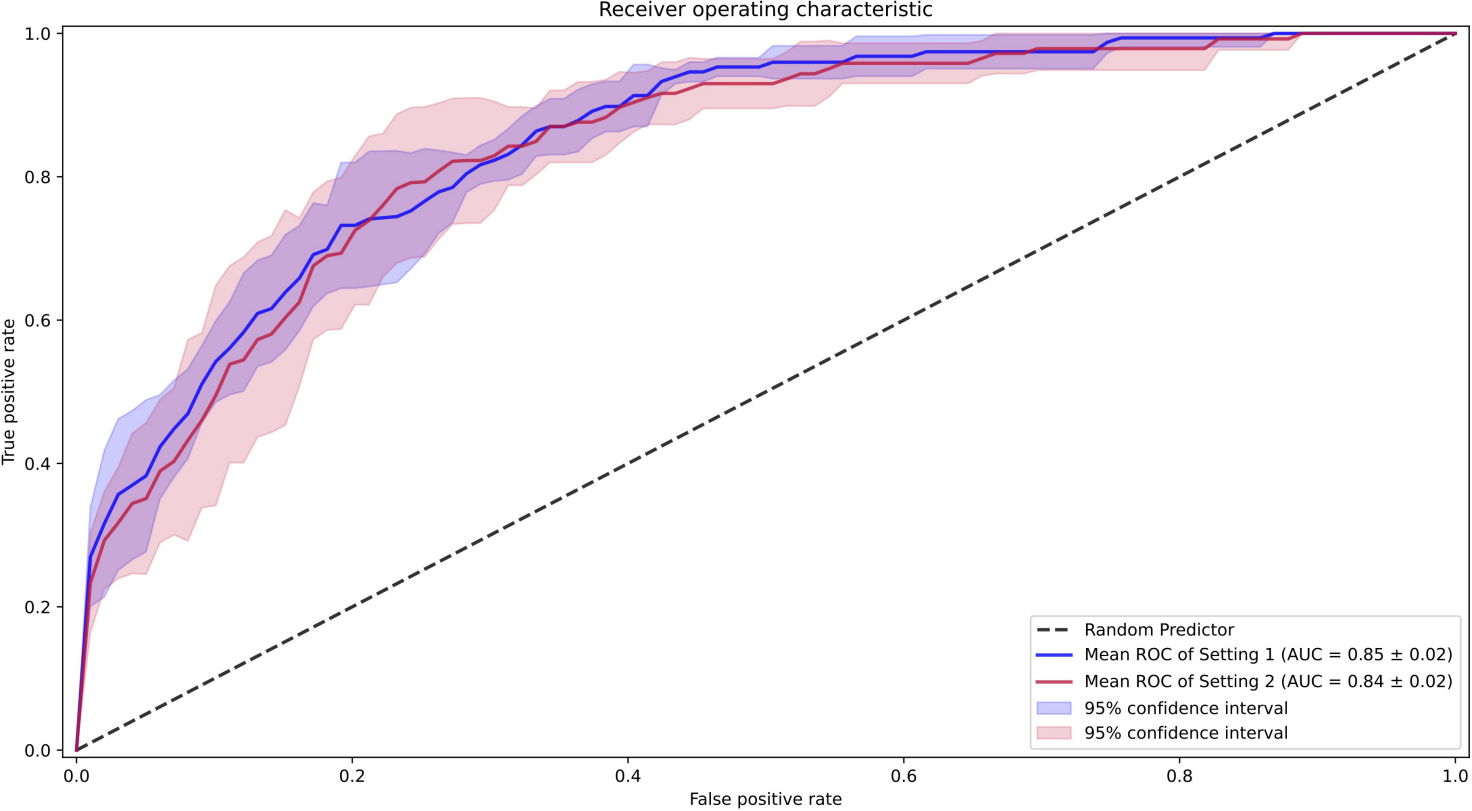
Receiver operating characteristic curves for the performance of the AI model. Setting 1 for with supraventricular ectopy (SVE) events and Setting 2 for without SVE events.

We first examined the performance of our AI model in both settings, where we assessed all the data, including those with SVE events (setting 1) and without SVE events (setting 2). For both settings, all folds were kept consistent with the same compositions of each patient, with only SVE events removed in setting 2. The AUROC (18) of setting 1 was 0.85 (0.83–0.87) and that of setting 2 was 0.84 (0.82–0.86), as shown in Figure 4. Additionally, the sensitivity, specificity, positive predictive value, negative predictive value, F1 score, and F2 score for both settings are shown in Table 1. To determine the optimal score cutoff of the classifiers, we chose the cutoff that maximized the sum of the sensitivity and specificity.

**Table 1:** Performance evaluation of our artificial intelligence model for settings 1 and 2.

	**Sensitivity**	**Specificity**	**PPV**	**NPV**	**F1 score**	**F2 score**
Setting 1	0.85 (0.79–0.92)	0.72 (0.63–0.82)	0.32 (0.28–0.37)	0.97 (0.96–0.98)	0.47 (0.42–0.51)	0.63 (0.61–0.66)
Setting 2	0.84 (0.79–0.90)	0.72 (0.65–0.82)	0.34 (0.27–0.40)	0.97 (0.96–0.98)	0.47 (0.41–0.53)	0.64 (0.60–0.68)
SVE burden	0.40 (0.39–0.40)	0.88 (0.88–0.88)	0.30 (0.30–0.30)	0.92 (0.92–0.92)	0.34 (0.34–0.34)	0.37 (0.37–0.37)

*SVE, supraventricular ectopy. The values in brackets refers to 95% confidence interval*.

**Figure 5:**
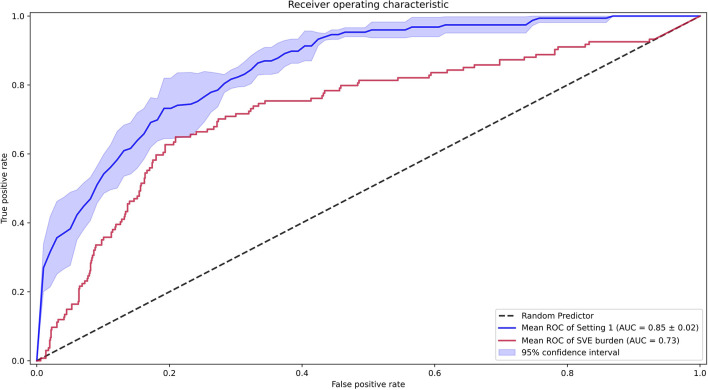
Receiver operating characteristic curves for the performance of the artificial intelligence model [setting 1 including supraventricular ectopy (SVE)], and SVE burden for Holter annotation.

Second, we compared the performance of our AI model against the SVE burden as an estimator of pAF probability. The AUROC of such an estimator was 0.73, and the AI model achieved significantly better performance than an SVE burden–based estimator in the pAF-detection task (Figure 5). For a more detailed comparison, we found an optimal score cutoff using the same method for the AI model and computed the sensitivity, specificity, positive predictive value, negative predictive value, F1 score, and F2 score as shown in Table 1.

### Differences in Nighttime and Daytime Ambulatory Holter Monitoring

**Figure 6:**
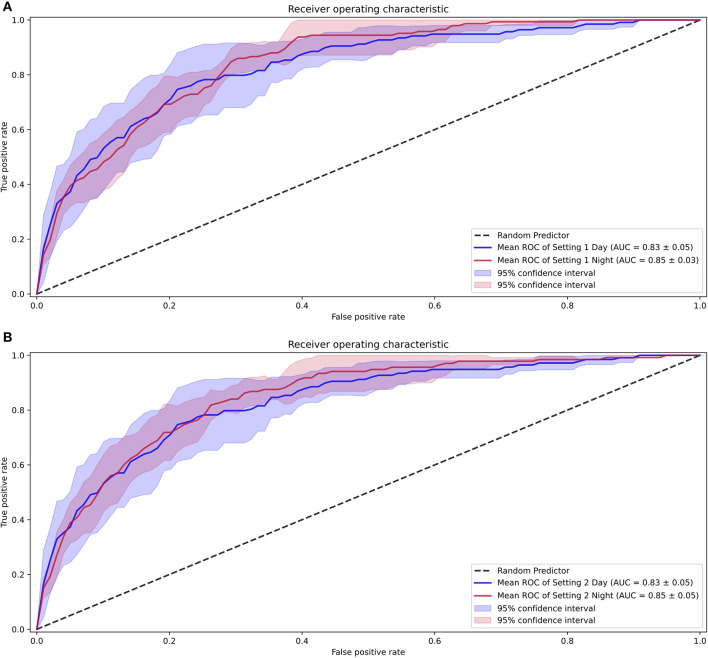
Receiver operating characteristic curves for the differences seen with diurnal periods. **(A)** Performance with setting 1. **(B)** Performance with setting 2.

In the analysis considering diurnal periods, the model intended for daytime analysis was trained on daytime data and the nighttime settings. As a result, the AUROCs for daytime were 0.83 (0.78–0.88) for setting 1 and 0.83 (0.78–0.88) for setting 2, while the AUROCs for nighttime were 0.85 (0.82–0.88) for setting 1 and 0.85 (0.80–0.90) for setting 2. The comparison between these settings is shown in Figure 6.

### Analysis of Model Sensitivity According to Time From Holter Monitoring to AF Diagnosis

Using the cutoff (T) of 3 months, the detection rate of pAF with the AI model for >3 months was 94.6% and that for <3 months was 91.8%. When T was 6 months, the detection rate for >6 months was 93.6% and that for <6 months was 92.2%. When T was 12 months, the detection rate for >12 months was 95.5% and that for <12 months was 92.0%. Finally, when T was 36 months, the detection rate for >36 months was 100% and that for <36 months was 92.0%.

## Discussion

This study shows that the described AI model, when using 24-h ambulatory continuous Holter monitoring without AF recordings, can identify occult pAF. The performance of our AI model outperformed the performance of the Holter exam with an SVE burden to identify pAF within the same database. Subgroup analysis according to the diurnal period, nighttime settings showed more favorable performance compared to daytime recordings. Finally, there was little performance difference between the time differences of AF diagnosis and Holter exam duration <12 months.

This study, to our knowledge, was the first to investigate pAF with AI using a continuous ambulatory rhythm monitoring system. The efforts in screening AF remain ongoing to aid in treatment decision-making or stroke prevention. Ambulatory Holter monitoring is an easy-to-use, non-invasive method that can detect even asymptomatic events owing to continuous recording. However, the detection rate of new-onset AF using 24-h Holter monitoring alone is low at 2.4–6.0% of patients (19). Prolonged monitoring with an implantable recorder detects more episodes but is invasive and costly. Recently, opportunistic intermittent ECG monitoring using remote rhythm sampling has been suggested, and intermittent ECG monitoring is significantly more likely to identify AF, (20, 21) although it also requires more extended monitoring periods and patient cooperation. David et al. reported that SVE burden on routine 24-h Holter was a strong independent predictor of prevalent subclinical AF (22). According to our study result, the performance of our AI model outperfromed that of the SVE burden to indentify pAF. Therefore, our study result can suggest that who will need intensive AF screening by filtering out those most likely to have AF.

Recent AI-driven research reported use of an AI-enabled ECG algorithm for identifying AF and predicting incident AF (23, 24). These studies used 12-lead, 10-s ECGs. In our study, we used 24-h continuous and ambulatory Holter monitoring to train the AI model. Some important points should be noted. The Holter monitor is a continuous and ambulatory recording system. It can represent diurnal variation and rhythm differences according to physical activity. For instance, Dilaveris et al. reported that P-wave duration, area, and P–R interval showed a significant circadian variation (25). Both P-wave duration and P–R interval were longer during the nighttime than the daytime. Our study results demonstrated that the performance of the AI model using nighttime recordings was more suitable for identifying occult AF than daytime recordings. Slowing heart rate and longer P-wave duration might make it possible to find the more subtle P-wave changes. We could not explain which factor influenced the identification of AF, the so-called black box. One hypothesis is that discrete structural changes such as fibrosis that precede atrial enlargement or phenotype change lead to ECG changes. A deep neural network can detect these subtle changes that the rule-based methods cannot confirm. Also, these changes are predicted to appear on the ECG for at least 12 months. Our study results showed little performance difference between the time differences of AF diagnosis and Holter exam duration between cutoff durations of 3, 6, and 12 months. However, when the duration was more than 36 months, the detection rate increased. These results may support our hypothesis.

While deep neural networks are potent for classification and prediction tasks for high-dimensional data, they must consume a massive amount of data to reach their true potential (26). Holter monitor–based ECG recordings, however, usually are challenging to collect in large numbers due to the difficulty faced in measurement processes. In addition, long sequences compared to standard 12-lead ECG recordings render Holter-based ECG datasets extremely high in dimension but low in number. Due to such characteristics of Holter data, harvesting the power of a deep neural network is necessary but, at the same time, it is difficult to train. Because classical machine learning algorithms are known to work better than deep neural networks when used in situations with limited amounts of training data points, we designed a two-step learning process to exploit the advantages of both classical machine learning algorithms and deep neural networks (27). Specifically, we have used CNN to encode 7-s-long ECG segments into a latent space by training the CNN to detect pAF at a segment-wise level. After 24-h ECG sequences were mapped into the latent space defined by the CNN, we trained a GBM to account for information underlying the entire sequence obtained from the original Holter recordings. This way, the models could consider both the underlying regularity encoded within individual short-time ECG segments and time-related information between the segments recorded from a single patient. As an ablation study, we have also evaluated pAF detection performance at segment-wise level. In these experiments, we only used CNN part of our full model taking out the GBM part. The performances of these experiments were much lower than entire sequence of original Holter recordings were considered. For both setting 1 and setting 2, AUROC came out to be 0.68 which is much lower than 0.85 and 0.84 of the original experiments. From this ablation study, we concluded that using the entire Holter recording sequence rather than short sequence is the key element in detecting pAF.

### Limitation

There are some limitations to our study. We used 3-lead ECGs with V1, V5, and III, instead of 12-lead ECGs for analysis. Although we did not use all 12 leads, some reports show that the P-wave terminal force on lead V1 reflects the atrial cardiomyopathic process (28). Therefore, it would have been sufficient to reflect some P-wave changes in the analysis. Furthermore, our study is thought to be a cornerstone of single-lead ECG AI analysis. In this regard, we could not perform external validation. The 12-lead ECG was achieved in a standard maneuver; however, Holter leads vary depending on the channel location, making them incompatible. To compensate for absence of external validation, we chose 5-fold cross validation to better estimate the average performances of other hypothetical datasets drawn from true distribution (29). Further, our study contains relatively small numbers of patients. However, previous studies using 12-lead ECGs performed their analyses with a 10-s signal, and our study analyzed 24-h ECG signals. Thus, it is believed that we gathered enough data for analysis. In addition, ambulatory continuous Holter monitoring contains more information than the data from a resting 12-lead ECG. Finally, we did not analyze subgroups with baseline characteristics, such as age or sex. The incidence of AF increases with age. If the age variable were used for analysis, it would have acted as a bias.

## Conclusion

We have demonstrated that an AI can identify occult pAF using 24-h ambulatory Holter monitoring achieved during sinus rhythm, and the performance of our AI model was better than the performance using the SVE burden in the Holter exam. Analysis using Holter monitoring can reflect the diurnal variation and some differences according to the level of physical activity. Further investigations will be necessary to confirm the performance in clinical practice.

## Data Availability Statement

The original contributions presented in the study are included in the article/supplementary material, further inquiries can be directed to the corresponding author.

## Ethics Statement

The studies involving human participants were reviewed and approved by Institutional Review Board of Eunpyeong St. Mary's Hospital. Written informed consent for participation was not required for this study in accordance with the national legislation and the institutional requirements.

## Author Contributions

JYK contributed to study design, data acquisition, data analysis, data interpretation, and writing of the report. KK contributed to data analysis, data interpretation, and writing of the report. YT and MC contributed to data analysis and data interpretation. S-JP, K-MP, YO and JSK contributed to critical revision of the report. YL contributed to study design, data acquisition, data analysis, and data interpretation. S-WJ contributed to study design, data acquisition, and critical revision of the report. All authors contributed to the article and approved the submitted version.

## Conflict of Interest

KK, YT, MC, and YL were employed by VUNO Inc. The remaining authors declare that the research was conducted in the absence of any commercial or financial relationships that could be construed as a potential conflict of interest.

## Publisher's Note

All claims expressed in this article are solely those of the authors and do not necessarily represent those of their affiliated organizations, or those of the publisher, the editors and the reviewers. Any product that may be evaluated in this article, or claim that may be made by its manufacturer, is not guaranteed or endorsed by the publisher.
